# Plasma methylated *HIST1H3G* as a non-invasive biomarker for diagnostic modeling of hepatocellular carcinoma

**DOI:** 10.3389/fmed.2025.1571737

**Published:** 2025-04-02

**Authors:** Weiwei Zhu, Huifen Wang, Yudie Cai, Jun Lei, Jia Yu, Ang Li, Zujiang Yu

**Affiliations:** ^1^Department of Infectious Diseases, The First Affiliated Hospital of Zhengzhou University, Zhengzhou, China; ^2^Precision Medicine Center, Gene Hospital of Henan Province, The First Affiliated Hospital of Zhengzhou University, Zhengzhou, China

**Keywords:** hepatocellular carcinoma, DNA methylation, *HIST1H3G*, early diagnosis, prognosis

## Abstract

**Background:**

DNA methylation carrying epigenetic aberrations could potentially serve as a non-invasive tool for revolutionizing cancer diagnosis and monitoring. Here, we comprehensively evaluated the diagnostic value of plasma methylated *HIST1H3G*, and constructed diagnostic and prognostic models aimed at facilitating early detection and improving the prognosis of hepatocellular carcinoma (HCC).

**Methods:**

The level of *HIST1H3G* promoter methylation in HCC tissues was evaluated based on the UALCAN database, followed by validation through serum samples collected from HCC patients. We recruited 205 participants, encompassing 70 HCC patients, 79 liver cirrhosis (LC) patients, 46 hepatitis patients and 10 HCC patients before and after treatment with either transarterial chemoembolization (TACE) or radiofrequency ablation (RFA). Analysis of plasma *HIST1H3G* was performed using methylation-specific quantitative polymerase chain reaction (qPCR). Diagnostic and prognostic prediction models were formulated using the random forest algorithm, and the performance of these models was rigorously evaluated through receiver operating characteristics curve (ROC) analysis.

**Results:**

The methylation level of *HIST1H3G* was markedly elevated in both HCC tissues and plasma samples derived from HCC patients. *HIST1H3G*, PIVKA-II, total bilirubin (TBIL) and age were selected as the optimal markers and were included in the development of a diagnostic model. This model demonstrated superior accuracy in distinguishing HCC from high-risk populations, outperforming alpha-fetoprotein (AFP) in both the training cohort consisting of LC patients and the validation cohort comprising hepatitis patients. Additionally, *HIST1H3G* and albumin (Alb) were chosen to establish a prediction model for early HCC diagnosis, and this model exhibited a remarkable ability to identify early HCC. Furthermore, our prognostic prediction model proved effective in predicting the prognosis and survival outcomes of HCC patients.

**Conclusion:**

Together, we identified and validated a diagnostic model that incorporated methylated *HIST1H3G* and clinically applicable serological indicators in HCC. The findings of our study established a pivotal foundation for the development of a non-invasive approach to identification and management in HCC.

## 1 Introduction

Hepatocellular carcinoma (HCC), the most common type of liver cancer, ranks as the third leading cause of cancer-related mortality globally, primarily attributed to liver cirrhosis (LC) resulting from chronic hepatitis virus infection, alcohol consumption, and fat accumulation (1–3). The asymptomatic nature of HCC in its early stages often results in patients being diagnosed at an advanced stage, with a dismal 5-year survival rate below 30% (4). Early detection holds immense potential in enabling timely treatment and improving survival rates. Unfortunately, there has been little success in developing effective serum-based screening methods for HCC (5, 6). Therefore, there remains a great need for minimally invasive, early detection methods to allow timely stratification of patients to appropriate therapies.

Among the various screening methods considered, liquid biopsy based on DNA methylation has emerged as a promising non-invasive diagnostic tool in clinical applications (7, 8). DNA methylation, a key epigenetic modification, plays a crucial role in transcriptional regulation of genes and maintaining the stability of the genome. Aberrant DNA methylation arises when a methyl group (CH_3_) is added to a cytosine base within a cytosine-phosphate-guanine (CpG) dinucleotide, triggering the deregulated transcription and activation of oncogenic pathways (9). In recent years, numerous efforts to detect cancer methylation have demonstrated that a significant proportion of genes exhibit aberrant DNA methylation patterns in cancerous tissues (10). Consequently, several methylated markers have been validated and approved for clinical use in cancer detection (11–13), including plasma methylated SEPT9 testing for colorectal cancer (14, 15). However, there is a paucity of validated methylation markers available for HCC. Furthermore, studies aimed at identifying HCC methylation biomarkers have often focused on advanced HCC patients and healthy individuals as controls, limiting their widespread application as routine screening tools in high-risk populations, such as patients with LC and chronic hepatitis. Therefore, it is imperative to conduct comprehensive profiling of chronic hepatitis, LC, and HCC patients to accurately identify early-stage HCC cases within these high-risk groups.

In this study, we identified *HIST1H3G* as a DNA methylation biomarker of HCC. We also compared the diagnostic efficiency of *HIST1H3G* with existing approaches, including AFP and PIVKA-II, in discriminating HCC patients from high-risk individuals with LC and chronic hepatitis using quantitative polymerase chain reaction (qPCR) analysis of plasma samples. Notably, we established a diagnostic prediction model employing plasma samples from 79 LC patients and 70 HCC patients. This model was then independently validated in a cohort comprised of 46 chronic hepatitis plasma samples, as well as 20 plasma samples collected from 10 HCC patients prior to and following treatment with either transarterial chemoembolization (TACE) or radiofrequency ablation (RFA). Additionally, leveraging the aforementioned 70 HCC plasma data, we further developed a prediction model specifically tailored for early HCC diagnosis, drawing upon the Barcelona Clinic Liver Cancer (BCLC) stage classification. Furthermore, a prognostic prediction model was constructed utilizing the same samples from HCC patients. Herein, we reported a diagnostic prediction model based on a DNA methylation biomarker to improve or complement current detection strategies, thereby enabling more precise prognostic stratification of HCC patients in clinical practice.

## 2 Methods

### 2.1 Participant information

Between December 2022 and October 2023, a total of 225 patients were recruited from the First Affiliated Hospital of Zhengzhou University, with 205 patients ultimately meeting the inclusion criteria. All patients with HCC were diagnosed in accordance with the “Diagnostic and Therapeutic Criteria for Primary Liver Cancer (2022 Edition)” issued by the Ministry of Health of China. LC was diagnosed based on imaging findings (CT or MRI). Chronic hepatitis patients were identified as those experiencing abnormal hepatic function indicators and digestive system symptoms, such as fatigue and anorexia, for a duration exceeding 6 months. All patients met the following criteria: (1) complete case data; (2) age between 18 and 80 years; (3) no history of malignant tumor in other organs.

A comprehensive set of clinical characteristics were gathered for each enrolled patient, including age, gender, alanine aminotransferase (ALT) levels, aspartate transaminase (AST) levels, total bilirubin (TBIL), AFP, PIVKA-II, neutrophil-to -lymphocyte ratio (NLR), platelet (PLT) count, prothrombin time (PT), blood ammonia levels, tumor size, tumor number, and BCLC staging. The BCLC staging system, recognized internationally as the gold standard for HCC clinical staging, demonstrates core superiority through its multidimensional integrated assessment framework and seamless linkage to therapeutic decision-making. These data were meticulously collected through the Hospital Electronic System. All laboratory examinations were conducted by the Department of Clinical Laboratory at the First Affiliated Hospital of Zhengzhou University. The study was approved by the Ethics Committee of the First Affiliated Hospital of Zhengzhou University, Zhengzhou, China (2023-KY-0425-002). Written informed consent of each patient was obtained.

### 2.2 Sample collection and storage

A total of 10 mL of venous blood was collected using cell-free DNA storage tube (CoWin Bioteck, Jiangsu, China). Plasma samples were isolated by repeated centrifugation at 1,500 g for 10 min at room temperature, and stored at −80°C for subsequent cfDNA extraction.

### 2.3 Methylation assay

We obtained data on the promoter methylation of *HIST1H3G* in HCC tissues from UALCAN database (available at: http://ualcan.path.uab.edu), an interactive web portal for performing in-depth analyses of TCGA gene expression data (16). The screening conditions were: “Gene: *HIST1H3G*”; “TCGA dataset: Liver hepatocellular carcinoma”; “Links for analysis: Methylation”; “Profile based on: Sample types, Individual cancer stages, and Tumor grade.”

The diagnostic kit designed for *HIST1H3G* methylation utilized qPCR detection in human plasma samples. BGI Genomics (Shenzhen, China) supplied all the necessary kits. The assay was conducted in strict accordance with the manufacturer’s instructions. Briefly, cell-free DNA (cfDNA) was extracted from plasma samples and underwent bisulfite conversion, followed by qPCR detection. ACTB (β-actin) was utilized as an internal control, serving as a benchmark for assessing both the quantity of cfDNA present in the samples and the efficiency of the PCR amplification process. For results to be deemed valid, the ACTB cycle threshold (Ct) must be below 34.8. Furthermore, a *HIST1H3G* Ct value below 45 was interpreted as a positive result, indicating the presence of methylation in the *HIST1H3G* gene.

### 2.4 Model construction

Methylation marker *HIST1H3G* and clinical parameters were applied to the diagnostic and prognostic models by random forest algorithm. Probability of disease (POD) index represented the ratio between the count of randomly generated decision trees that predicting a sample as “HCC” and that of a control. The selected optimal markers were employed to compute the POD index for both the diagnostic and prognostic models. For the assessment of these models, the receiver operating characteristic (ROC) curve was generated using R version 3.3.0 with the pROC package, and the area under this curve (AUC) served as an indicator of the ROC performance.

### 2.5 Statistical analysis

Statistical analyses were carried out utilizing SPSS Version 21 (SPSS, Chicago, IL, United States), GraphPad Prism version 8 (GraphPad Software, San Diego, CA, United States) software tools and R language (version 3.3.0). The Wilcoxon rank sum test was employed to compare continuous variables across the two groups. Differences among the three groups were assessed using one-way ANOVA. Categorical variables were compared using Fisher’s exact test. *p*-values <0.05 were considered statistically significant.

## 3 Results

### 3.1 Patient characteristics

**Figure 1:**
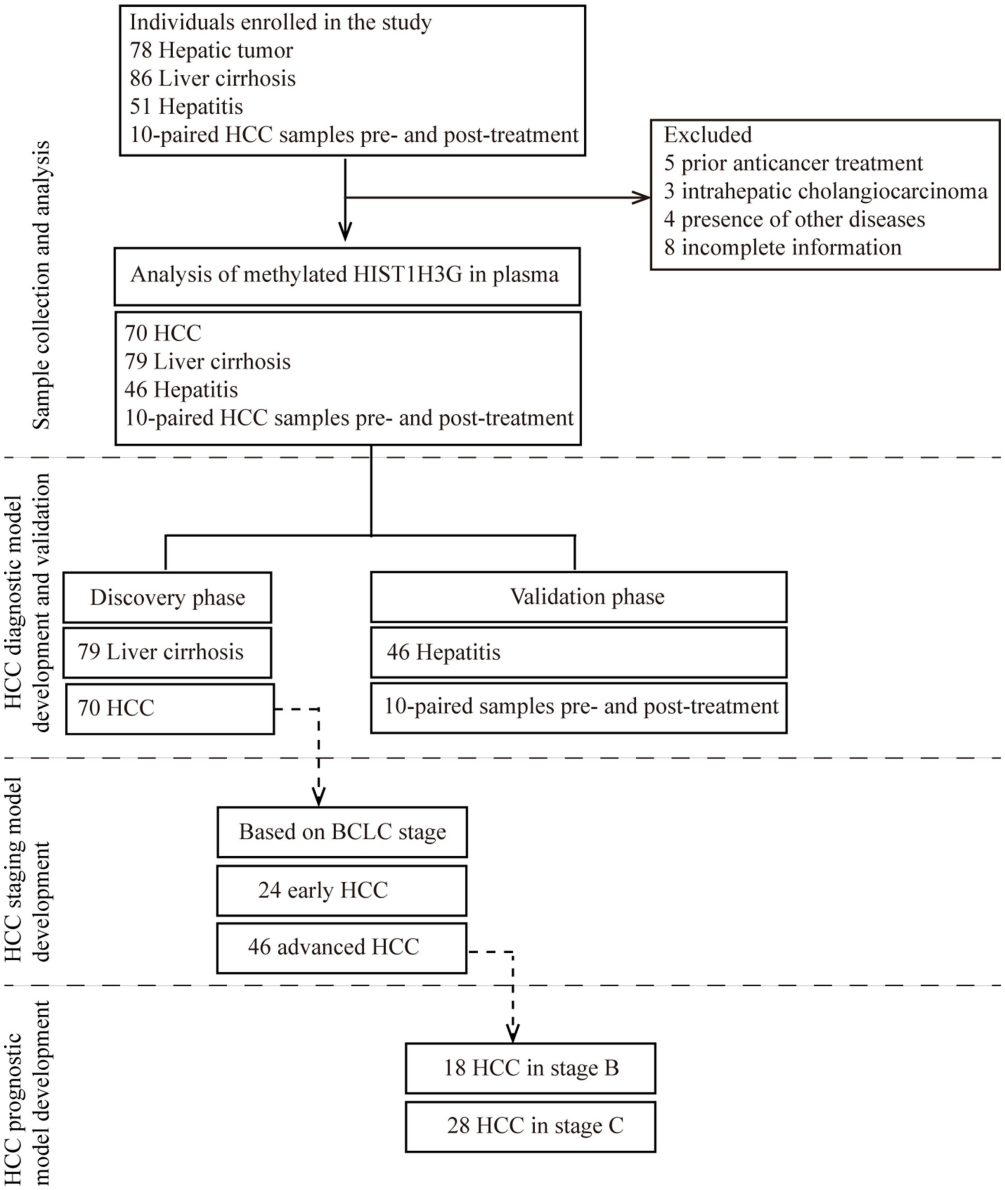
Study design. The major objective was to develop a non-invasive tool for early detection of HCC based on methylated *HIST1H3G*. The training phase comprised of 79 liver cirrhosis serum samples and 70 HCC serum samples was to construct a diagnostic prediction model. This model underwent independent validation within a cohort comprised of 46 hepatitis plasma samples, as well as 20 plasma samples collected from 10 HCC patients prior to and following treatment with either TACE or RFA. Due to limited sample size, only hepatitis and HCC patients were available in the validation phase. Moreover, using the aforementioned 70 HCC plasma samples, we developed a prediction model specifically designed for early HCC diagnosis based on the BCLC staging criteria. Additionally, a prognostic prediction model was constructed utilizing the HCC samples of 18 stage B and 28 stage C. HCC, hepatocellular carcinoma; BCLC stage, Barcelona Clinic Liver Cancer stage.

We consecutively enrolled a total of 225 hospitalized patients. After a thorough exclusion process, 20 patients were excluded, leaving us with 70 HCC patients, 79 LC patients, 46 chronic hepatitis patients, and 10 HCC patients who were recruited prior to undergoing either TACE or RFA procedure and followed up for biomarkers 1 month post-treatment. Subsequently, the 205 patients who met the inclusion criteria were divided into the training phase and the validation phase. Employing plasma samples from 79 LC patients and 70 HCC patients, a diagnostic prediction model was constructed. This model underwent independent validation within a cohort comprised of 46 chronic hepatitis plasma samples, as well as 20 plasma samples collected from 10 HCC patients prior to and following treatment with either TACE or RFA. Moreover, using the aforementioned 70 HCC plasma samples, we developed a prediction model specifically designed for early HCC diagnosis based on the BCLC staging criteria. Among these 70 samples, 24 samples categorized as stage 0-A were defined as early-stage HCC, while 18 samples in stage B and 28 samples in stage C were collectively classified as late stage HCC. This approach ensured greater precision and reliability in diagnosing early HCC. Additionally, a prognostic prediction model was constructed utilizing the HCC samples of stage B and stage C. The study design was depicted in Figure 1.

The clinical characteristics of the participants are summarized in Table 1. The mean age of the HCC group (56.0 ± 9.7 years) was significantly higher compared to the LC group (51.0 ± 12.3 years) and the hepatitis group (47.0 ± 13.5 years). Male patients were more prevalent in the HCC group (77.1%) than in the hepatitis group (52.2%), while there was no significant difference in the male-to-female ratio between the HCC and LC groups (77.1% vs. 78.5%). HBV or HCV infection was more common in the HCC group than in the chronic hepatitis group (90.0% vs. 43.4%), but no significant difference was observed between the LC and HCC patients (79.8% vs. 90.0%). Serum levels of AFP and PIVKA-II were greatly elevated in HCC patients compared to those with LC and hepatitis (both *p* < 0.05). The mean ΔCt value of *HIST1H3G* was notably lower in HCC patients compared to both LC and hepatitis patients (both *p* < 0.05). NLR was higher in the HCC group than in the LC and hepatitis groups (both *p* < 0.05). TBIL levels in HCC group were significantly reduced compared to LC group (*p* = 0.012) and hepatitis group (*p* = 0.024). Serum concentrations of ALT and AST were markedly decreased in HCC patients compared to those with hepatitis (all *p* < 0.05). The mean PLT count in HCC patients was significantly higher than in LC patients (*p* = 0.001) but lower than in hepatitis patients (*p* = 0.001). Additionally, the concentrations of PT was also substantially lower in HCC patients compared to those with LC (*p* < 0.05). However, there were no statistically significant differences in the median levels of TP, Alb, Hb, and blood ammonia among the groups (all *p* > 0.05).

**Table 1:** Clinical characteristics of the enrolled participants.

Clinical characteristics	Hepatitis (*n* = 46)	LC (*n* = 79)	HCC (*n* = 70)	*p*-values (hepatitis vs. HCC)	*p*-values (LC vs. HCC)
Age (year)	47.0 (13.5)	51.0 (12.3)	56.0 (9.7)	<0.001	0.011
Gender					
Female	22 (47.8%)	17 (21.5%)	16 (22.9%)	0.005	0.844
Male	24 (52.2%)	62 (78.5%)	54 (77.1%)		
Aetiological factors					
Viral hepatitis (HBV/HCV)	20 (43.4%)	63 (79.8%)	63 (90.0%)	<0.001	0.084
Non-viral hepatitis	26 (56.5%)	16 (20.3%)	7 (10.0%)		
AFP (0–8.87 ng/mL)	300.6 (1142.7)	34.1 (102.1)	27591.6 (107446.3)	0.027	0.010
PIVKA-II (0–40 mAU/mL)	46.1 (78.4)	71.6 (374.4)	13968.9 (25422.4)	<0.001	<0.001
*HIST1H3G* (ΔCt value)	12.0 (5.1)	13.0 (4.6)	5.7 (5.3)	<0.001	<0.001
Tumor size (cm)					
≤3	—	—	40 (57.1%)		
3–5	—	—	30 (42.9%)		
No. of tumor					
Single	—	—	29 (41.4%)		
Multiple	—	—	41 (58.6%)		
BCLC stage					
A	—	—	28 (40.0%)		
B	—	—	17 (24.3%)		
C	—	—	25 (35.7%)		
Cirrhosis					
Yes	0 (0%)	79 (100%)	66 (94.3%)		
No	46 (100%)	0 (0%)	4 (5.7%)		
ALT (<40 U/L)	212.8 (295.8)	56.4 (104.3)	43.0 (43.0)	0.001	0.657
AST (<40 U/L)	168.1 (173.1)	84.3 (134.8)	69.3 (66.9)	0.002	0.766
TBIL (μmol/L)	79.6 (109.7)	70.4 (100.1)	30.7 (63.3)	0.024	0.012
TP (g/L)	64.0 (7.3)	62.2 (6.9)	64.3 (6.9)	0.805	0.069
Alb (35–55 g/L)	37.5 (5.4)	34.4 (6.0)	36.0 (5.7)	0.164	0.085
Hb (120–160)	151.6 (149.6)	112.9 (22.9)	126.6 (19.5)	0.079	0.265
PLT (83–303 × 10^9^/L)	181.8 (73.6)	96.8 (65.0)	135.8 (73.5)	0.001	0.001
NLR	2.3 (2.8)	2.7 (2.2)	3.7 (3.2)	0.012	0.019
PT (9–14 s)	13.2 (5.8)	15.8 (4.6)	13.4 (2.0)	0.996	0.000
Blood ammonia (16–60 μmol/L)	44.4 (21.8)	46.9 (23.9)	48.9 (26.0)	0.330	0.619

LC, liver cirrhosis; HCC, hepatocellular carcinoma; HBV, hepatitis B virus; HCV, hepatitis C virus; AFP, alpha-fetoprotein; PIVKA-II, protein induced by vitamin K absence or antagonist-II; BCLC stage, Barcelona Clinic Liver Cancer stage; ALT, alanine aminotransferase; AST, aspartate aminotransferase; TBIL, total bilirubin; TP, total protein; Alb, albumin; Hb, hemoglobin; PLT, platelet; NLR, neutrophil to lymphocyte ratio; PT, prothrombin time.

### 3.2 The methylation level of *HIST1H3G* in HCC tissues and plasma

**Figure 2:**
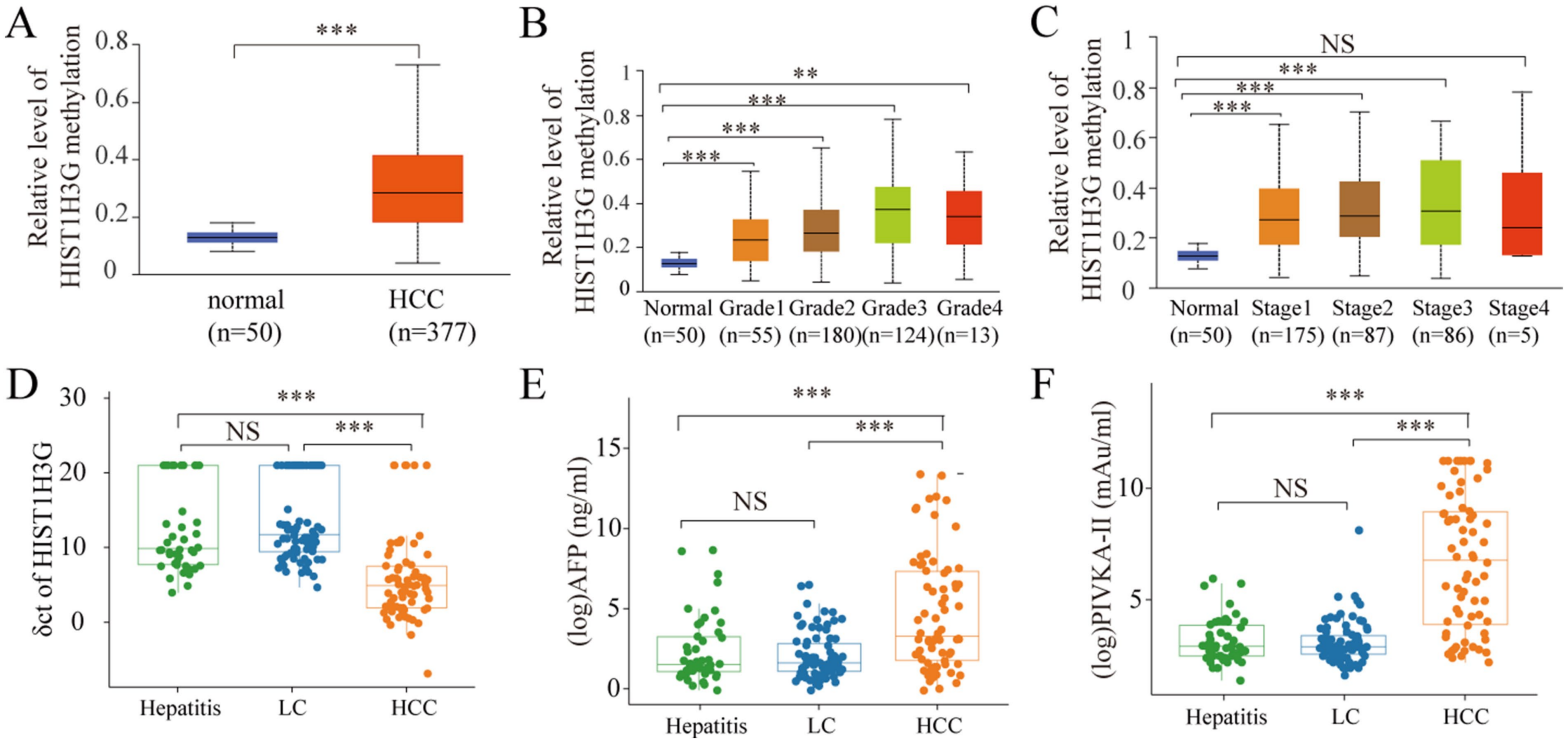
Analysis of methylated *HIST1H3G* in tissue and plasma, and analysis of AFP and PIVKA-II in serum. **(A–C)** Promoter methylation levels in HCC, different grades and stages obtained from UALCAN database. **(D)** ΔCt values of *HIST1H3G* in enrolled groups. **(E,F)** Serum levels of AFP and PIVKA-II in enrolled groups. The values plotted were derived through the application of logarithmic transformation to the AFP and PIVKA-II levels. HCC, hepatocellular carcinoma; LC, liver cirrhosis; NS, no significance; AFP, alpha-fetoprotein; PIVKA-II, protein induced by vitamin K absence or antagonist-II.

Firstly, based on UALCAN database, we identified the expression level of methylated *HIST1H3G* in HCC tissues. Our findings revealed a obviously higher methylation level of *HIST1H3G* in 377 HCC tissues versus 50 adjacent normal tissues (Figure 2A). We also observed that elevated methylated *HIST1H3G* expression correlated with advanced TNM stage (Figure 2B) and low differentiation grade (Figure 2C). This indicated that hyper-methylation of *HIST1H3G* played a crucial role in the molecular pathogenesis and progression of HCC.

For a more comprehensive study, we obtained plasma samples from 46 chronic hepatitis patients, 79 LC patients, and 70 HCC patients. The mean ΔCt value of *HIST1H3G* in the HCC group was markedly decreased compared to both the hepatitis and LC groups (Figure 2D). The lower ΔCt values of *HIST1H3G* observed in HCC patients suggested significantly higher DNA methylation levels when compared to those with LC and hepatitis. Furthermore, our results corroborated the elevation of serum AFP and PIVKA-II in the HCC group (Figures 2E,F). These results suggested that methylated *HIST1H3G* could potentially serve as a biomarker for HCC.

### 3.3 The diagnostic value of plasma methylated *HIST1H3G* in patients with HCC

**Figure 3:**
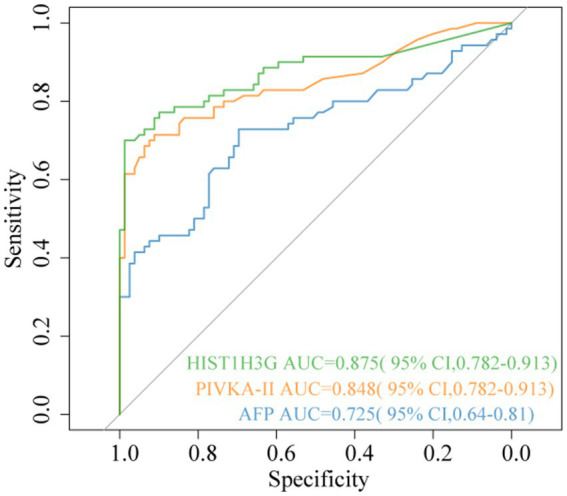
Diagnostic outcomes for serum *HIST1H3G*, PIVKA-II and AFP in the diagnosis of HCC. AUC (*HIST1H3G*) > AUC (PIVKA-II) > AUC (AFP). AUC, the area under the ROC curve; AFP, alpha-fetoprotein; PIVKA-II, protein induced by vitamin K absence or antagonist-II.

To assess the diagnostic value of methylated *HIST1H3G*, the hepatitis and LC groups were combined into a single non-HCC group. ROC curve was constructed to evaluate the performance of *HIST1H3G*. Additionally, AFP and PIVKA-II, which are commonly employed as serum-based biomarkers for HCC screening in clinical practice, were evaluated to assess their diagnostic accuracy. As presented in Figure 3, *HIST1H3G* demonstrated the highest area under the ROC curve (AUC) at 0.875. The AUCs for AFP and PIVKA-II were 0.725 and 0.848, respectively. Our results showed that *HIST1H3G* exhibited excellent diagnostic accuracy in differentiating HCC from non-HCC cases, closely followed by PIVKA-II, and both outperformed AFP.

Each biomarker exhibited distinct patterns of serum level distribution and range. We conducted an analysis of the correlation coefficients to ascertain statistically relationships between the biomarkers. The correlation coefficient between AFP and *HIST1H3G* was 0.4 (*p* < 0.05) (Supplementary Figure 1A), whereas the correlation between PIVKA-II and *HIST1H3G* was 0.58 (*p* < 0.05) (Supplementary Figure 1B). Furthermore, a correlation coefficient of 0.59 was observed between AFP and PIVKA-II (*p* < 0.05) (Supplementary Figure 1C). The findings indicated a weak correlation between *HIST1H3G* and both AFP and PIVKA-II, implying that *HIST1H3G* may be utilized independently as an adjunctive diagnostic tool for HCC patients.

### 3.4 Construction of the diagnostic prediction model for HCC

To demonstrate the diagnostic value of the methylated *HIST1H3G* for HCC, we developed a diagnostic prediction model that could specifically identify HCC individuals from high-risk populations, including those with LC and hepatitis. The POD index was calculated using the identified optimal markers in both the training and the validation cohorts.

**Figure 4:**
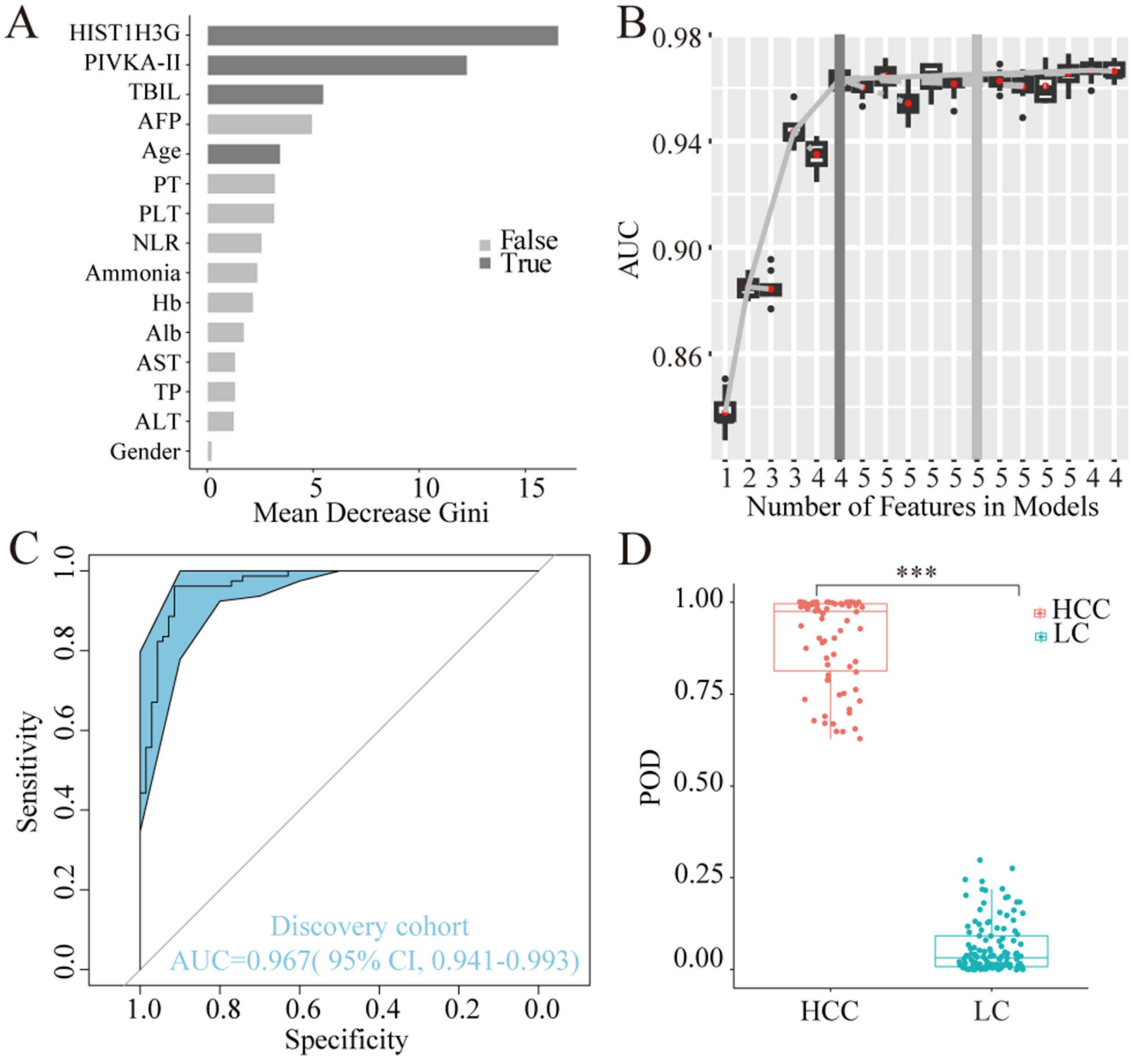
Construction of the diagnostic prediction model for HCC by random forest model. **(A)** *HIST1H3G*, PIVKA-II, TBIL, and age were selected as the optimal marker set. **(B,C)** The POD index achieved an AUC value of 0.967 with 95% CI of 0.941 to 0.993 between HCC and LC cohorts in the discovery phase. **(D)** The POD value was significantly increased in the HCC samples versus the LC samples (*p* < 0.001). AUC, the area under the ROC curve; POD, probability of disease; HCC, hepatocellular carcinoma; LC, liver cirrhosis; PIVKA-II, protein induced by vitamin K absence or antagonist-II; TBIL, total bilirubin; AFP, alpha-fetoprotein; PT, prothrombin time; PLT, platelet; NLR, neutrophil to lymphocyte ratio; Hb, hemoglobin; Alb, albumin; AST, aspartate aminotransferase; TP, total protein; ALT, alanine aminotransferase.

Utilizing the training dataset comprising 70 HCC and 79 LC samples, we employed a random forest model in the discovery phase. The analysis revealed *HIST1H3G*, PIVKA-II, TBIL, and age as the most discriminatory markers (Figure 4A). Incorporating these four markers yielded the highest AUC value for the model (Figure 4B). In the training phase, our diagnostic prediction model exhibited an impressive AUC of 0.967, with a 95% CI ranging from 0.941 to 0.993, effectively separating the HCC and LC cohorts (Figure 4C). The POD value was significantly elevated in HCC samples compared to LC samples (*p* < 0.05, Figure 4D). These findings strongly suggested that our diagnostic prediction model, which was based on the selected markers, held considerable promise for accurately identifying HCC patients within the LC cohort.

### 3.5 Validation diagnosis and supplemental performance of the classifier for HCC

**Figure 5:**
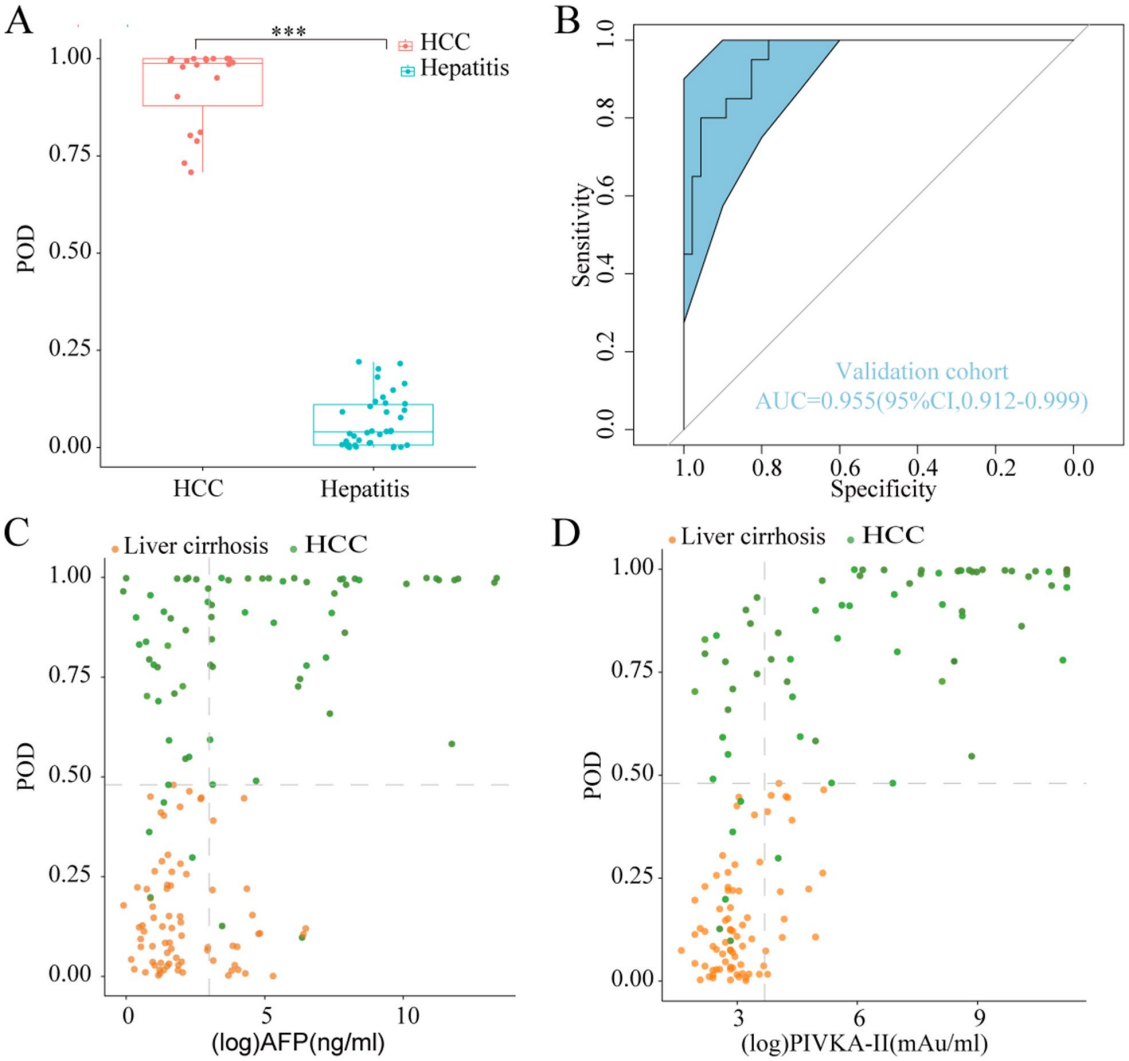
Validation diagnosis and the complementary role for the diagnosis of AFP-negative and PIVKA-II-negative HCC patients. **(A)** The POD value was significantly increased in the HCC samples versus the hepatitis samples in the validation phase (*p* < 0.001). **(B)** The model performed robustly in validation phase, which achieved an AUC value of 0.955 (95% CI 0.912 to 0.999) between HCC and hepatitis cohorts. **(C,D)** Performance of the model in the diagnosis of AFP-negative and PIVKA-II-negative HCC patients. POD, probability of disease; AUC, area under the curve; HCC, hepatocellular carcinoma; AFP, alpha-fetoprotein; PIVKA-II, protein induced by vitamin K absence or antagonist-II.

During the validation phase, a total of 46 hepatitis samples, along with 20 samples obtained from 10 HCC patients prior to and following TACE or RFA procedure, were employed to authenticate the diagnostic efficacy of the model in detecting HCC. Importantly, the classification accuracy exhibited remarkable concordance between the training and validation datasets, attesting to the effectiveness of the modeling process and the minimal occurrence of over-fitting (Table 2). Furthermore, the mean POD value was significantly elevated in the 20 samples from the 10 HCC patients compared to the 46 control samples (*p* < 0.05, Figure 5A). Remarkably, the model achieved an AUC value of 0.955 (95% CI 0.912 to 0.999) when distinguishing between HCC and hepatitis cohorts (Figure 5B), thereby validating its substantial diagnostic potential for HCC.

**Table 2:** Performance of the POD for the diagnosis of HCC in training and validation cohort.

Cohort	AUC (95% CI)	Sensitivity (%)	Specificity (%)	PPV (%)	NPV (%)
Training cohort	0.967 (0.941–0.993)	91.43	96.20	95.52	92.68
Validation cohort	0.955 (0.912–0.999)	90.0	82.0	69.23	95.0

POD, probability of disease; AUC, the area under the ROC curve; HCC, hepatocellular carcinoma; PPV, positive predictive value; NPV, negative predictive value.

We further explored the complementary role of our model for AFP and PIVKA-II in the diagnosis of HCC. The diagnostic value of the classifier was evaluated in HCC patients who were initially undetected by AFP or PIVKA-II testing, using cutoff values established in clinical practice (AFP at 20 ng/mL, PIVKA-II at 40 mAU/mL). Our classifier exhibited a remarkable capacity in distinguishing AFP-negative HCC patients from those with LC (Figure 5C). Additionally, the classifier’s performance in discriminating PIVKA-II-negative HCC from LC remained noteworthy (Figure 5D). These results demonstrated that our diagnostic model greatly improved the ability of AFP and PIVKA-II to differentiate HCC from high-risk patients.

### 3.6 Abilities of *HIST1H3G* in identifying early HCC

**Figure 6:**
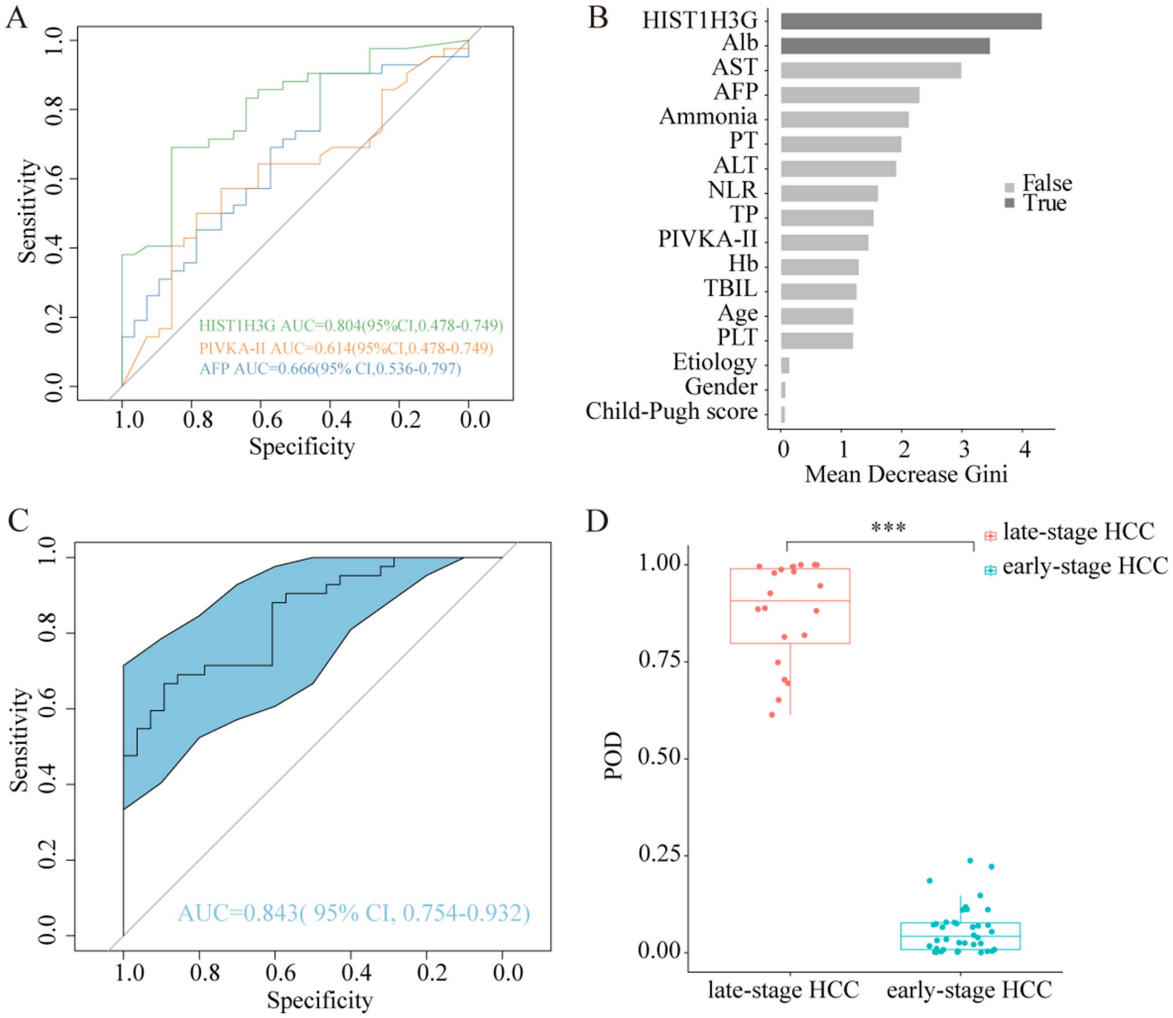
Abilities of *HIST1H3G* in identifying early HCC. **(A)** Diagnostic outcomes for serum *HIST1H3G*, PIVKA-II and AFP in the diagnosis of early HCC. AUC (*HIST1H3G*) > AUC (AFP) > AUC (PIVKA-II). **(B)** *HIST1H3G* and Alb were selected as the optimal marker set. **(C)** The POD index achieved an AUC value of 0.843 with 95% CI of 0.754 to 0.932 between early-stage HCC and late-stage HCC cohorts. **(D)** The POD value was significantly increased in the late-stage HCC samples versus the early-stage HCC samples (*p* < 0.001). AUC, the area under the ROC curve; POD, probability of disease; HCC, hepatocellular carcinoma; Alb, albumin; AST, aspartate aminotransferase; AFP, alpha-fetoprotein; PT, prothrombin time; ALT, alanine aminotransferase; NLR, neutrophil to lymphocyte ratio; TP, total protein; PIVKA-II, protein induced by vitamin K absence or antagonist-II; Hb, hemoglobin; TBIL, total bilirubin; PLT, platelet.

To assess the diagnostic potential of *HIST1H3G* in the detection of early HCC, we utilized the aforementioned 70 HCC plasma samples to validate the reliability of this methylation biomarker. According to the BCLC staging criteria, 24 samples from the 70 were categorized as stage 0-A, representing early-stage HCC, while 18 samples in stage B and 28 samples in stage C were collectively grouped as late-stage HCC. A ROC analysis was conducted to compare the diagnostic performance of *HIST1H3G* with AFP and PIVKA-II in aiding the diagnosis of early HCC. The AUC for *HIST1H3G* was superior to both AFP and PIVKA-II (Figure 6A), indicating its promising potential as a biomarker for the early detection of HCC.

Similarity, we developed a prediction model specifically designed for early HCC diagnosis, with *HIST1H3G* and Alb chosen as the optimal markers (Figure 6B). The average POD value was significantly elevated in the 24 patients with advanced HCC compared to the 46 patients with early HCC (Figure 6D). Furthermore, the POD achieved an AUC value of 0.843 (95% CI 0.754 to 0.932) when discriminating between the two groups (Figure 6C). These results underscored the robust diagnostic performance of our prediction model in identifying patients with early HCC, indicating its potential as a powerful tool for early detection.

### 3.7 Construction of prognostic prediction model for HCC

**Figure 7:**
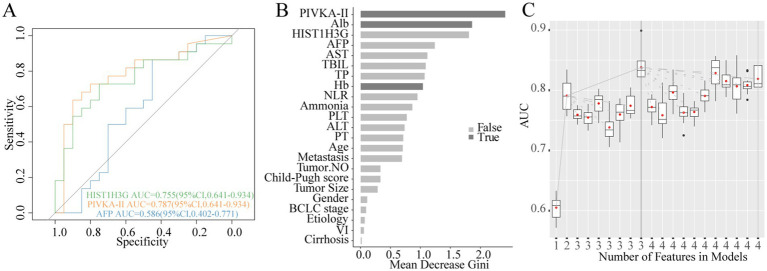
Construction of prognostic prediction model for HCC. **(A)** Diagnostic outcomes for serum *HIST1H3G*, PIVKA-II and AFP in predicting the survival status of patients with advanced HCC. AUC (PIVKA-II) > AUC (*HIST1H3G*) > AUC (AFP). **(B,C)** PIVKA-II, Alb, and Hb were selected as the optimal marker set. AUC, the area under the ROC curve; PIVKA-II, protein induced by vitamin K absence or antagonist-II; Alb, albumin; AFP, alpha-fetoprotein; AST, aspartate aminotransferase; TBIL, total bilirubin; TP, total protein; Hb, hemoglobin; NLR, neutrophil to lymphocyte ratio; PLT, platelet; ALT, alanine aminotransferase; PT, prothrombin time; VI, vascular invasion; HCC, hepatocellular carcinoma.

We conducted a thorough analysis to explore the relationship between methylated *HIST1H3G* expression and the prognosis of 46 patients with advanced HCC (18 in stage B, 28 in stage C), excluding stage 0-A due to the small number of deaths. Subsequently, these patients were stratified into deceased and surviving groups based on their outcomes. Our results, presented in Figure 7A through ROC curves, demonstrated that PIVKA-II and *HIST1H3G* exhibited superior sensitivity than AFP in assessing the survival outcomes of HCC patients. Moreover, PIVKA-II, ALB, and Hb emerged as the optimal markers, playing a crucial role in predicting the survival status of patients with advanced HCC (Figures 7B,C). These data indicated that *HIST1H3G* served not only as a valuable biomarker but also as an aid for clinicians in predicting the prognosis of patients with advanced HCC.

Based on previous research findings, we observed that *HIST1H3G*, AFP, PIVKA-II, ALB, and Hb held significant predictive value in determining the staging or prognosis of HCC. Therefore, a total of 10 paired before and after procedure serum samples obtained from HCC patients were analyzed to further identify their ability in evaluating treatment response. Within the cohort, consisting of six participants in the surviving group and four participants in the deceased group, comparative analysis revealed that *HIST1H3G*, AFP, PIVKA-II, ALB, and Hb exhibited non-significant alterations pre- and post-treatment in the surviving cohort (all *p* > 0.05, Supplementary Figures 2A–E). It was worth mentioning that *HIST1H3G* increased post-treatment in surviving group, albeit without statistical significance compared to pre-treatment (Supplementary Figures 2A,F). Analogous trends were observed within the deceased group (Supplementary Figures 2F–J).

## 4 Discussion

HCC remains a rather challenging and devastating liver disease at the global level (1). Early detection is the most effective way to reduce HCC mortality. AFP is the well-known and widely used biomarker for HCC. However, the observation that 80% of patients with small HCC showed no increase in AFP level (17), which indicated that AFP was not optimal for the detection of early HCC. DNA methylation, being a crucial epigenetic modification, typically manifests as an early event in carcinogenesis, presenting considerable potential as biomarkers for the early detection, staging of cancer and prognosis (10, 18). Therefore, we undertook the present work to achieve a comprehensive classification of HCC and establish tools for HCC prognostic and therapeutic efficacy assessment. To our knowledge, this is the inaugural investigation utilizing DNA methylation marker *HIST1H3G* in model establishment and validation for HCC.

Currently, the identification of HCC methylation biomarkers typically studied in healthy individuals serving as controls, thus constraining its potential for widespread application as a routine screening tool in high-risk populations. Patients suffering from cirrhosis and chronic liver disease remained at a significantly elevated risk for HCC occurrence (2). Additionally, non-alcoholic steatohepatitis (NASH), often associated with metabolic syndrome or diabetes mellitus, is emerging as the fastest growing etiology of HCC, especially in the West (19, 20). Given this, we decided to incorporate these high-risk populations as our control groups, rather than relying solely on healthy individuals. Consequently, we conducted a comprehensive analysis on patients with chronic hepatitis, LC and HCC, aiming to accurately detect early HCC cases among these high-risk groups. This approach was more likely to represent the distribution of the population in actual clinical practice in China.

The emerging field of liquid biopsy is pioneering innovative diagnostic approaches for cancer and other diseases (21). Cancer-specific DNA methylation patterns have been investigated as feasible biomarkers in various cancers. In a large-scale clinical validation study, Gao et al. (22) successfully established a model for the early detection and localization of six distinct cancer types affecting the colorectum, esophagus, liver, lung, ovary, and pancreas, utilizing cfDNA methylation technology. Numerous promising methylation-based screening models for HCC have been put forward. Luo et al. (23) identified 2,321 DNA methylation biomarkers of tissue samples with high throughput DNA bisulfite sequencing and developed a HCC screening model, which achieved a sensitivity of 86% and specificity of 98% in the training cohort and a sensitivity of 84% and specificity of 96% in the independent validation cohort. Through integrated analyses of RNA-sequencing and DNA methylation data, Long et al. (24) constructed diagnostic and prognostic models using two DNA methylation-driven genes in HCC and achieved satisfactory performances. Kisiel et al. (25) reported a panel of six methylated DNA markers based on sequencing of tissues in phase II, which yielded a best-fit AUC of 0.96 (95% CI, 0.93–0.99) when detecting early-stage HCC in high-risk populations. In our study, we identified *HIST1H3G* as a DNA methylation biomarker by qPCR in plasma samples, and developed diagnostic and prognostic models based on methylated *HIST1H3G*.

*HIST1H3G* is a member of the histone H3 family, encoding a replication-dependent histone that plays a crucial role in maintaining the structure of nucleosomes within chromosome fibers in eukaryotes. Histone modifications regulate chromatin structure and gene expression by adding or removing specific chemical groups to histones. For instance, histone acetylation is typically associated with gene activation, while histone methylation may either suppress or activate gene expression, depending on the specific methylation site (26, 27). In this study, we determined that DNA methylation level of *HIST1H3G* was overexpressed both in HCC tissues and serum. The specific increase of *HIST1H3G* in HCC suggested that it could be a potential marker of HCC. Moreover, the diagnostic ability of *HIST1H3G* was superior than that of AFP and PIVKA-II. Consistent with our findings, it has been reported that *HIST1H3G* was involved in the development of glioblastoma, acute lymphoblastic leukemia and cervical cancer (13, 28, 29). Specifically, significant increased *HIST1H3G* in methylation was confirmed in lung adenocarcinoma based on a genome-wide methylation screening (30). Besides, Zhang et al. (31) presented that *HIST1H3G* was upregulated and considered key methylation marker with higher AUC values for osteoporosis. Although the mechanism and biological function of *HIST1H3G* hypermethylation in HCC tumorigenesis have not been reported, our data demonstrated that *HIST1H3G* could still be a potential DNA marker in HCC.

An efficient screening assay must exhibit a sufficiently high degree of specificity to minimize false positive rates and to avoid unnecessary anxiety and medical expenditures among individuals without HCC. To construct a highly accurate model, random forest algorithm was used to select optimal markers in our study. Our diagnostic model, which integrated *HIST1H3G*, PIVKA-II, TBIL, and age, effectively distinguished patients with HCC from those with LC and chronic hepatitis across the training and validation cohorts. The sensitivity of our classifier for HCC was comparable to surveillance with ultrasound (32), the current recommendations for HCC screening. Meanwhile, the model also exhibited potential diagnostic capability in both AFP-negative and PIVKA-II-negative groups, underscoring its complementary role in HCC diagnosis alongside AFP and PIVKA-II. These findings demonstrated that our model had strong classification potential for discriminating HCC from LC and chronic hepatitis individuals, and held promise as a non-invasive diagnostic tool for HCC.

In addition, we found that hypermethylation of *HIST1H3G* significantly outperformed than AFP and PIVKA-II in distinguishing early-stage HCC from all stage HCC patients. Although serum AFP is the most clinically utilized biomarker for HCC, its performance in detecting early-stage HCC remains far from optimal (17). As many as 40% of HCC cases across all stages in cirrhotic patients demonstrate normal serum AFP levels, and detection rates for early-stage HCC may be as low as 30% according to previous studies (6, 33). Recently, PIVKA-II proposed to serve as a complementary or alternative diagnostic tool to AFP for HCC, while its diagnostic performance in detecting early-stage HCC was notably influenced by the underlying liver disease etiology (6). Our study also helped to demonstrate the unsatisfactory performance of AFP and PIVKA-II for early-stage HCC. In contrast, our model showed high sensitivity and specificity for early HCC detection. More importantly, high methylation level of *HIST1H3G* showed comparable ability with PIVKA-II in forecasting mortality in HCC patients, even though *HIST1H3G* was not selected for model construction by the random forest model. Our study lays a foundation for the possibility of using *HIST1H3G* methylation as diagnostic and prognostic biomarker for HCC in serum samples of patients.

Despite the significance of our prediction model, several limitations might impede the interpretation of our results. Firstly, the hepatitis/LC group had shorter median ages than the HCC group in both the discovery and validation cohorts, which may have influenced the biomarker levels. An age-matched cohort might reduce the potential selection bias. Secondly, different benign liver diseases served as control group, LC were included in training phase, while hepatitis patients with different etiologies were included in validation phase. The same disease population in both cohorts would be helpful to improve the model for achieving the most optimal performance. Thirdly, the number of enrolled HCC patients in the validation cohort was relatively small, especially the data both before and after treatment were used for analysis. Additional different stages of HCC participants would be helpful to validate the robustness of our model. Fourthly, the reliability of our prognostic model was constrained by a relatively short clinical follow-up and a limited number. In the forthcoming period, we plan to evaluate the expression levels and concordance of *HIST1H3G* in both tissues and serum of HCC patients, and conduct multi-center studies with a substantial sample size to verify the robustness of its diagnostic power. Afterwards, we will establish a sensitive technique to identify the existence of *HIST1H3G* in cfDNA for screening patients with HCC.

In conclusion, prediction models were meticulously developed and validated, utilizing a combination of a DNA methylation biomarker alongside clinically applicable protein markers and routine biochemical assessments. To the best of our knowledge, this is the first time that methylated *HIST1H3G* has been used in the construction of diagnostic and prognostic models for HCC patients. These models possessed significant predictive value for HCC, indicating their potential utility in prognosis and personalized medical treatment strategies. The findings of our study on HCC serve as a pivotal foundation for developing a non-invasive approach for cancer identification and management, with potential applications across a wide range of malignancies.

## Data Availability

The datasets presented in this study can be found in online repositories. The names of the repository/repositories and accession number(s) can be found in the article/Supplementary material.
