# Supplementary material for: Plasma methylated HIST1H3G as a non-invasive biomarker for diagnostic modeling of hepatocellular carcinoma
Source: Front Med (Lausanne). 2025 Apr 2;12:1571737. doi: 10.3389/fmed.2025.1571737 (PMC12000021; doi:10.3389/fmed.2025.1571737)
Supplement: Supplementary file 1 [file Table_1.docx]

Supplementary Material

# Supplementary Figures and Tables

## Supplementary Figures


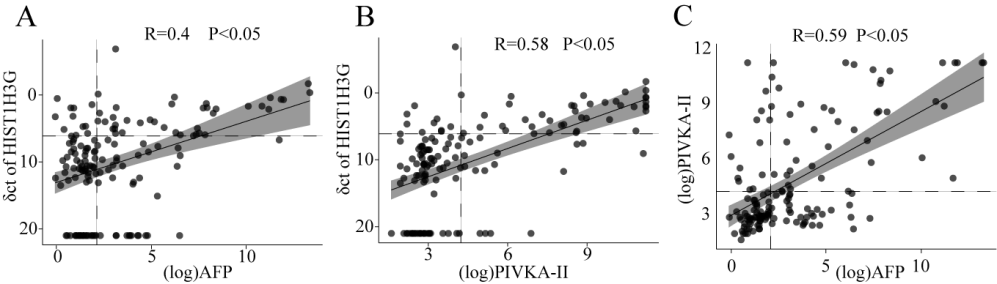


**Supplementary Figure 1.** Correlations between plasma HIST1H3G level and AFP level **(A)**, PIVKA-Ⅱ level **(B)**, and correlations between PIVKA-Ⅱ level and AFP level **(C)** using the non-parametric Spearman’s rank correlation analysis. The values plotted were derived through the application of logarithmic transformation to the AFP and PIVKA-Ⅱ levels.

Abbreviation: AFP, Alpha-fetoprotein; PIVKA-Ⅱ, protein induced by vitamin K absence or antagonist-II.


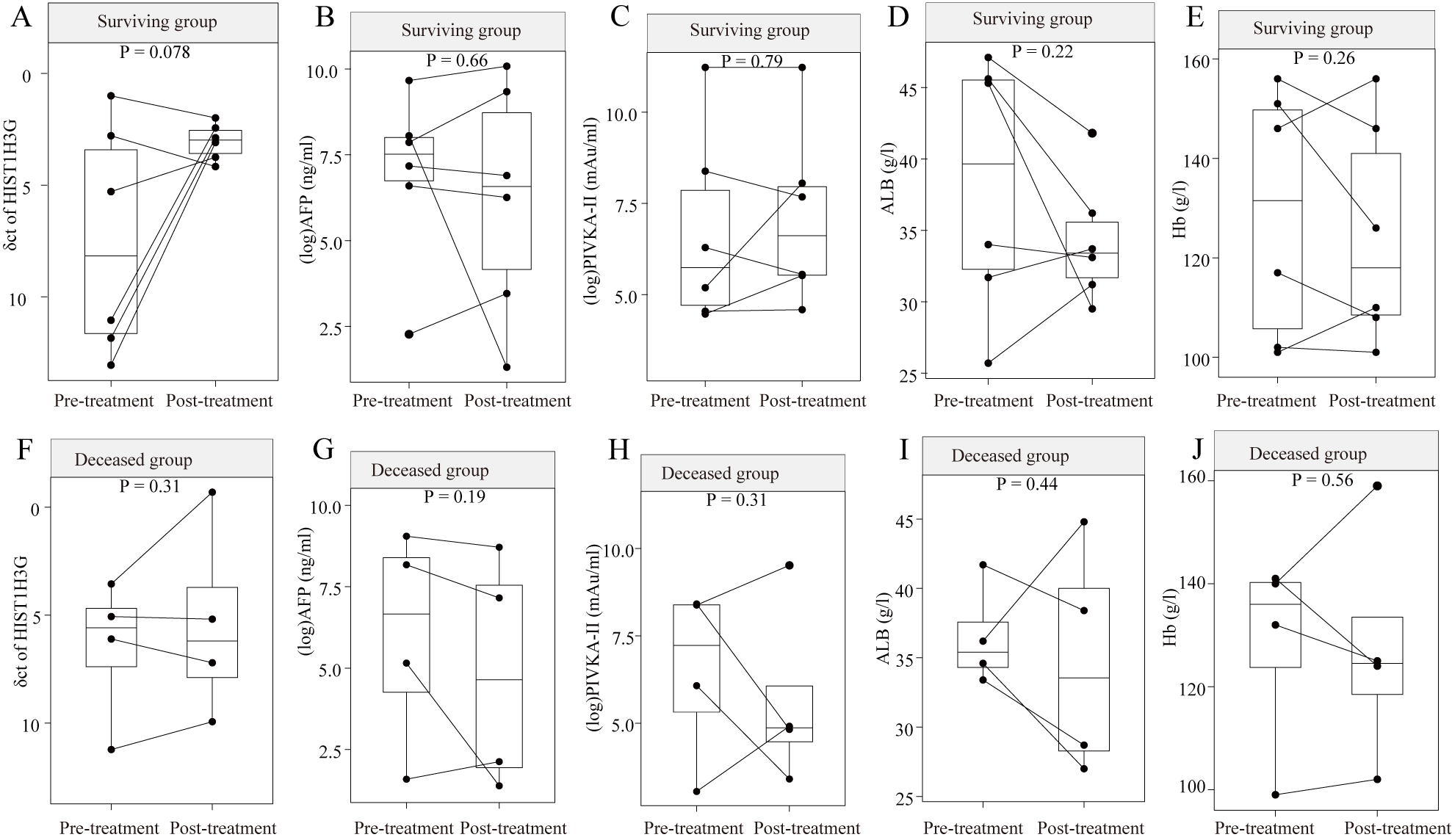


**Supplementary Figure 2.** Comparison of HIST1H3G, AFP, PIVKA-Ⅱ, ALB and Hb for HCC treatment response in the surviving group and deceased group. (A) HIST1H3G increased post-treatment in surviving group, albeit without statistical significance compared to pre-treatment. AFP, PIVKA-Ⅱ, ALB and Hb exhibited non-significant alterations pre- and post-treatment in the surviving cohort (B-E) and in the deceased group (F-J).

Abbreviation: AFP, Alpha-fetoprotein; PIVKA-Ⅱ, protein induced by vitamin K absence or antagonist-Ⅱ; Alb, albumin; Hb, Hemoglobin.
